# Wildlife Strike Risk Assessment in Several Italian Airports: Lessons from BRI and a New Methodology Implementation

**DOI:** 10.1371/journal.pone.0028920

**Published:** 2011-12-14

**Authors:** Cecilia Soldatini, Yuri Vladimir Albores-Barajas, Tomas Lovato, Adriano Andreon, Patrizia Torricelli, Alessandro Montemaggiori, Cosimo Corsa, Vyron Georgalas

**Affiliations:** 1 Department of Environmental Sciences, Informatics and Statistics, University Ca'Foscari of Venice, Venice, Italy; 2 Aeroporto di Venezia Marco Polo S.p.A. SAVE, Venice, Italy; 3 Stazione Romana Osservazione e Protezione Uccelli, Rome, Italy; 4 ENAC - Ente Nazionale Aviazione Civile, Rome, Italy; 5 CMCC – Centro Euro-Mediterraneo per i Cambiamenti Climatici, Bologna, Italy; University of Western Ontario, Canada

## Abstract

The presence of wildlife in airport areas poses substantial hazards to aviation. Wildlife aircraft collisions (hereafter wildlife strikes) cause losses in terms of human lives and direct monetary losses for the aviation industry. In recent years, wildlife strikes have increased in parallel with air traffic increase and species habituation to anthropic areas. In this paper, we used an ecological approach to wildlife strike risk assessment to eight Italian international airports. The main achievement is a site-specific analysis that avoids flattening wildlife strike events on a large scale while maintaining comparable airport risk assessments. This second version of the Birdstrike Risk Index (BRI2) is a sensitive tool that provides different time scale results allowing appropriate management planning. The methodology applied has been developed in accordance with the Italian Civil Aviation Authority, which recognizes it as a national standard implemented in the advisory circular ENAC APT-01B.

## Introduction

Wildlife, particularly birds, is increasingly present in human-modified habitats due to an increase of synanthropic species populations and to the process of habituation to anthropogenic resources that many species are undergoing [1], [2]. Many of these species present life histories that promote adaptation to urban environmental characteristics [3], [4], [5].

This factor, together with the increase of air traffic [6], has resulted in a worldwide increase in the number of wildlife strikes [6], [7]. To our knowledge, in peer reviewed ISI journals there are only four methods to perform a bird strike risk assessment [8], [9], [10], [11]. A more effective approach to the growing wildlife hazards for the air safety should involve an ecological based tool capable to deal with the species-specific characteristics present in airports, where 96% of the wildlife strikes occur [6].

There are two main components of a wildlife strike event: aircraft and wildlife. From the aircraft perspective, there is not much that can be done to avoid strikes because aircraft have a set route and speed for takeoff and landing and any change to these parameters may create more danger than reducing it. Nowadays, aircraft characteristics are fairly homogeneous on a geographical scale, while from the wildlife perspective, there is a much larger variability, both seasonal and geographical. For instance, some species are gregarious off the breeding period but become solitary/coupled during the breeding period. Generally, during the migration period, there is a higher richness and abundance along the migratory routes. Besides, after the breeding season, the inexperienced juveniles involved in collisions with aircraft may contribute significantly to the increase of wildlife strikes [12].

The high variability of biogeographic gradients poses a serious problem to wildlife strike risk assessment, as it is reflected in wildlife community composition. This means that airports present in the same geographical area may have differences in wildlife community composition due to differences in the environmental characteristics present in the immediate vicinity and, thus, different risks for wildlife strikes. Habituation to anthropic environments is a process sensitive to cultural dynamics [3], [13], therefore the same species may be at different stages of the process in different geographical areas [2], [14].

Human activities in the surrounding areas of airports are also crucial in determining the wildlife strike risk because they may attract numerous species that are hazardous for air navigation [15]. The Italian Civil Aviation Authority (hereafter ENAC) requires the environmental monitoring of an airport's surrounding area within a 13 km radius from the airport [16]. Furthermore, in Italy, the airport management authority is responsible for collecting and submitting data to ENAC of all wildlife strikes occurring within the airport's perimeter and up to a height of 300 ft [17].

Among the several methods to estimate wildlife strike hazard proposed [8], [9], [10], [11], some use an economic perspective [8], [11], while others use data collected on a national level [9]. The major problem with these approaches is that they may not reflect the characteristics of each individual airport, making comparisons between airports difficult. To render things even more complicated it often happens that the wildlife strike data available are incomplete because records from pilots may lack species information or carcasses may be lost [18]. Thus, there is a general need for a standardized method that is easy to apply and statistically robust. Considering the differences in monitoring programs between airports, the risk assessment tool should work with different time series of data.

On a previous version of the Birdstrike Risk Index BRI [10], we proposed a method that takes into account the ecological characteristics of the bird communities resident in the airport area. In the first version of the index, the novel approach allowed to correlate some of the variables involved in the birdstrike, but since more data became available and airports inter-comparisons were recursively required, the need of an Index improvement appeared necessary.

In this paper, we propose a modified version of the Birdstrike Risk Index, BRI [10], here named BRI2, with the aim of introducing a more general applicability by improving the species categorization and testing the robustness of the group risk factor. A formal revision of the index structure was also carried out in order to enable the comparison of results among different airports.

## Results

An increasing trend in the number of flights per year (Table 1) was observed in the major part of the investigated airports for the period 2006–2010, contrarily to the high variability of wildlife strike tendency that appears to be largely influenced by site-specific conditions. The linear regression analysis between airport traffic and wildlife strikes resulted to be significant only for airport D, with a correlation coefficient equal to 0.89.

**Table 1:** Summary statistics of the linear regression on the number of flights and wildlife strike events in time, and between the number of flights and strikes events.

Airport	Flights			Wildlife strikes			Flights vs WildlifeStrikes	
	Beta	R^2^	P	Beta	R^2^	P	Spearman's rho	P
A	0,675	0,455	0,211	−0,414	0,171	0,586	−0,100	0,873
B	0,856	0,734	0,064	0,811	0,658	0,096	0,564	0,322
C	0,993	0,985	0,001	0,957	0,916	0,011	0,700	0,188
D	0,225	0,051	0,716	−0,073	0,005	0,907	0,894	0,041
E	0,741	0,549	0,152	0,852	0,727	0,066	0,200	0,747
F	−0,933	0,615	0,021	0,817	0,668	0,091	0,300	0,624
G	−0,784	0,870	0,116	−0,148	0,022	0,812	0,700	0,188
H	0,231	0,053	0,709	0,589	0,347	0,296	0,616	0,269

**Figure 1:**
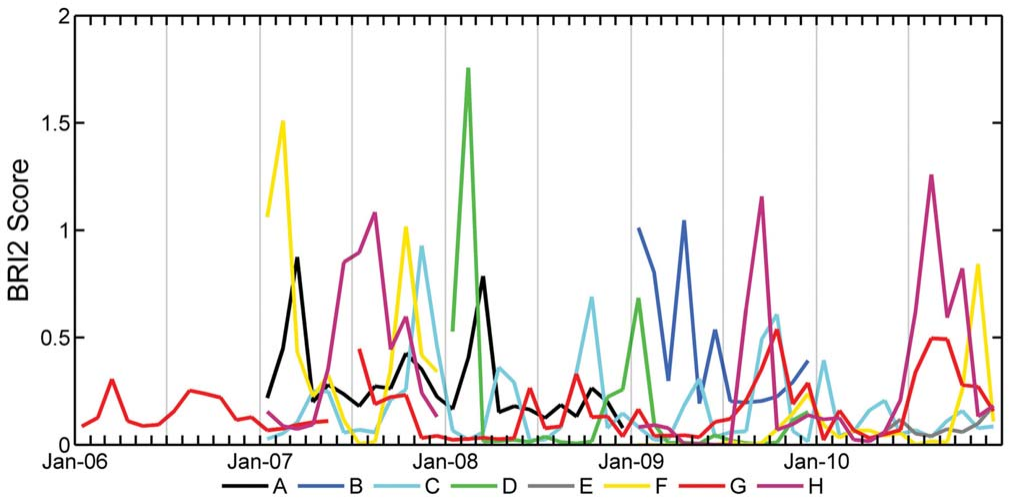
BRI2 scores for the eight investigated Italian airports in the period 2006 –2010.

The results obtained from the application of BRI2 to the eight investigated airports are depicted in Figure 1.

As expected, each airport presents different seasonal trends due to differences in wildlife community composition and their site-specific strike history. In airport G, where the longer time series of bird abundance is available, a seasonal trend with higher values in late summer months is clearly visible. This trend is attributable both to the first autumn migration movements which are associated to the large presence of hazardous groups 6, 7, and 12 (juveniles of kestrels and gulls and migratory species). As can be seen in Figure 1, a peak due to feral pigeons (*Columba livia*, a domestic form) foraging in late spring (May–June) during reproductive activity in nearby urban areas is present in airport E. A comparison of the BRI2 values for 2010 between airports G and E (∼20 km of distance) did not evidence a statistically significant correlation (R =  −0.49; p>0.05).

The statistical comparison of BRI2 values computed for airports F and H, which are 30 km apart, was not significant, being R =  −0.13 at the 95% confidence level. With both airports located in the same geographic region and near a major city, differences in the immediate vicinity surrounding environment influence the wildlife community's composition and abundance therefore producing different BRI2 scores. In particular, airport F shows higher BRI2 scores during the cold seasons, which are determined by the foraging movements of the flocking passerines group (group 15, principally starlings) from the city to the surrounding cropland areas. This group is characterized by high EOF_95_ and aggregation index values (EOF_95_  =  4; *Ag*_15_  =  40), in opposition to the same group values for the airport H, where specific EOF_95_ is much lower (EOF_95_  =  1) although aggregation index values are higher (*Ag*_15_  =  67). In fact, the main contribution in determining the risk peaks for the airport H, which is located between the city and the sea, is given by the group 7, 8 and 9 (in particular gulls and waders) that uses the area for roosting during the warmer period.

Among the 8 investigated airports, the highest wildlife strike risk is associated to the airport D, which belongs to the air traffic class 1. Such a result can be easily explained by considering that the wildlife strike risk history associated to the group of waders (group 9 here constituted mainly by lapwings *Vanellus vanellus*) is significantly higher than all the others groups, having a EOF_95_ equal to 2 and an aggregation index of 30 individuals.

The computed trends for the airports A and B showed a periodic peak in spring and autumn, respectively, while the results obtained for the airport C did not allowed to identify a clear pattern, as the experimental dataset was poor.

The analysis of BRI2 scores degradation due to the presence of an increasing number of undetermined values in the wildlife strike reporting lead to encouraging results.

It was possible to accept up to a 20% reduction of the strikes dataset for the airport G, before the BRI2 trend significantly degraded, as a consequence of a poor reliability of the Group Factor. In fact, while the first two comparisons did not result statistical significant (Wilcoxon, n = 12, p> 0.05 both tests), the comparison between the complete dataset and those containing more than 30% of undetermined data resulted statistically different (Wilcoxon, n = 12, p<0.05 all tests).

## Discussion

The increasing trend of wildlife strikes recorded worldwide in recent years [7] poses a serious threat to air traffic safety. In Italy, the same trend has been confirmed by the recorded wildlife strike reports [19]. Although air traffic increase on a global scale is undoubtedly responsible, when single airports are considered, other factors may contribute substantially to this increase, such as larger populations of synanthropic species or the presence of attractive sites near airports, such as landfills and fish culture ponds.

In all airports studied in the present work, apart from airport D, no significant correlations were found between the increase in air traffic and the number of wildlife strike events (Table 1). This indicates that the variation in the number of wildlife strike events do not reflect the sole increase of air traffic trend. It is therefore important to investigate the ecological and behavioral characteristics of wildlife communities present in airport areas.

A key aspect of the proposed index is the possibility to compare the risk level associated with wildlife presence, even if differences exist among site communities and surrounding environment information are missing. In particular, direct environmental information are neglected in the computation of BRI2, since they are assumed to be triggered by the local wildlife community composition. The recent introduction of more stringent safety protocols [17], [20], [21] and an increased awareness of stakeholders toward the wildlife strike problem has undoubtedly played a major role in the increase of wildlife strike reporting. Nevertheless, it remains difficult to accurately quantify the contribution of this “human factor” to the increased number of reported wildlife strikes.

Worldwide, an increased urbanization of many synanthropic species has been observed [3]. A well known example is the yellow-legged gull, *Larus michahellis* [4], [5], which has dramatically increased its breeding and wintering population during the last 20 years in Italy and Europe [22], [23]. This population increase seriously affects air safety since Gulls are commonly recognized as hazardous species worldwide [8]. In Italy, for instance, this species was the one most involved in wildlife strike incidents [24]. The European starling's population (*Sturnus vulgaris*) is also increasing in Italy, where different populations migrate or are resident depending on the latitude [25]. This species also behaves differently in winter, assembling in larger flocks in southern Italy. The variability shown by these two species is only an example of what can be expressed by a whole community at the local level. Therefore, a “risk coefficient” calculated on a national (or international) scale would flatten a species' hazardousness at the local level, preventing a site-specific risk assessment [8], [9].

The results obtained by applying the BRI2 algorithm on 8 Italian airports with an homogeneous distribution of air traffic characteristics are encouraging and allow a comparison between different airport sizes thus providing a site-specific evaluation of the wildlife strike risk. To our opinion, one of the main goals was met: BRI2 application renders comparison between different size-class airports possible even if wildlife monitoring data are non-homogenously collected and without the need to incorporate environmental characteristics information.

The BRI2 algorithm is being adopted as a standard by ENAC in order to perform a wildlife risk assessment (ENAC Advisory Circular APT-01B) at a national level. The elevated heterogeneity of the data collection methods and the limited extensions of the datasets used in the present manuscript does not permit a robust estimation of the risk associated with each group. The imminent standardization and improvement of the wildlife data collection methods in Italy (guidelines will be included in the ENAC Advisory Circular APT-01B) would hopefully contribute to the acquisition of high quality data series allowing a reanalysis of the index under the perspective of introducing confidences on its estimates.

Nonetheless our results show that there are different wildlife strike risk level trends for each airport (Figure 1). These trends can be explained at a site-specific level by the seasonal variation in local wildlife communities, thus allowing site-specific management planning.

The encouraging results obtained from the analysis of degradation introduced by different amounts of undetermined strike data may allow to better asses the reliability of index, especially when the quality of experimental data is poor. Under this perspective, the index can also be used to screen situations at an airport where safety protocols are not properly applied. However, a proper and complete monitoring program should be implemented to reasonably rely on the BRI2 scores.

The index was conceived as a tool capable of describing an airport specific wildlife strike risk, based upon historical trend of wildlife observations, in order to identify critical periods during the year. Therefore, the index is not meant to be a prognostic index since bird distribution throughout the years is unlikely predictable although it can be applied to assess specific theoretical risk scenarios.

Finally, the occurrence of changes in bird behavior during the migratory period [26], [27], would introduce a useful connection between local conditions and changes on a broader scale. For future research, it would be therefore important to integrate information on the birds migratory routes for the risk assessment process.

## Materials and Methods

ID numbers used for field work in airports:

- Cecilia Soldatini VCE airport N. 7205, TSF airport N.11273
- Yuri Albores-Barajas VCE airport N 8004, TSF airport N. 11274
- Lucio Panzarin VCE airport N. 7223, TSF airport N. 11275

### Data Collection

The wildlife presence data analyzed in the present manuscript were provided by eight Italian airports, under the agreement of an anonymous treatment of information. The data were collected by professional ornithologists or professionally trained airport ground staff (Bird Control Units) on an hourly basis during daylight or every 2–3 hours per day. The personnel were provided with a security pass from aerodrome operators for accessing the restricted areas. As site specific wildlife data were collected with heterogeneous sampling frequency, the average daily abundance for each species was used for the computation of the BRI2 index.

The aircraft movement data for each airport (in terms of flight numbers per month comprising both landings and takeoffs) were provided directly by the airport management authority. The airports were subdivided into 3 classes according to the yearly averaged Total Flight Number (TFN) registered in period 2003–2010 (Table 2): class 1: small-scale airport 1<TFN<50,000; class 2: medium-scale airport 50,001<TFN<99,999; class 3: large-scale airport TFN>100,000.

**Table 2:** List of investigated airports (ID letter), with the specific traffic size class, and the available time series extension for wildlife observations and strikes data.

Airport	Airport class	Wildlife data availability (years)	Wildlife strike Data availability (years)
A	1	2007–2008	2006–2010
B	1	2009	2006–2010
C	1	2007–2010	2006–2010
D	1	2008–2009	2006–2010
E	1	2010	2006–2010
F	2	2007, 2009–2010	2004, 2006–2010
G	2	2006–2010	2003–2010
H	3	2007, 2009–2010	2000–2010

The wildlife strike data were provided from the ENAC for the years 2006–2010 while strike data of the period prior to 2006 were provided directly from each airport authority. A summary of the wildlife abundance and strike data used in the present paper for each airport is reported in Table 2.

The eight studied airports are representative of the 37 present in Italy in terms of air traffic. On the basis of the information provided by the Italian Airport Association, a power analysis was performed to determine the reliability of the selected subsample of airports, obtaining a margin error of 0.094 at the 95% significance level. With regards to the three main traffic classes here considered, 5 out of 27 airports belong to class 1, 2 of 7 are in class 2, and 1 out of 3 is in class 3.

The overall amount of flights registered at the selected airports during the studied period (2006–2010) was equal to 4,870,158, while the number of wildlife strikes registered was 920. Similarly, the subset distribution for the total number of flights and wildlife strike events was representative of the entire Italian condition (a total of 2270 impacts), as confirmed by the power analysis with a margin of error of 0.65 at the significance level of 95%.

A descriptive analysis was carried out by means of linear regressions, in order to identify the presence of significant trends in the air traffic and impacts time series, as well as, direct relationships between the yearly number of flights and wildlife strike accidents.

### BRI2 index

The modifications here proposed to the BRI [10] are described in the next three points.

1. Group composition: two more groups are added to take into account strikes occurring with non-avian wildlife species. In particular, we introduced group 16 (mammals of small dimensions, weight under 10 kg) and group 17 (mammals of large dimensions, weight more than 10 kg). The groups composition proposed in Soldatini, et al. [10] is therefore extended as reported in Table 3. A full list of species associated with each group within the Italian territory can be found at the ENAC website (www.enac.gov.it).
2. Effect On Flight (EOF): in the previous publication [10], the maximum value of EOF recorded for a certain group of species was used in an effort to assume the worst event scenario, as long time series of strike data were unavailable. Presently, we propose and implement the use of the 95^th^ percentile (EOF_95_) instead of the previously proposed 100^th^ (EOF_max_). In fact, as long term series become available, the use of the 100^th^ percentile would overestimate the potential risk associated to a specific group. Furthermore, in an effort to adopt a standardized EOF classification, the same description used in the ICAO (International Civil Aviation Organization) Bird Strike Reporting Forms was here implemented. The new EOF severity scale is reported in Table 4.
3. BRI2 normalization: as the comparison between different traffic-scale airports was concerned, the BRI2 score was normalized toward the monthly number of flights in each airport.

**Table 3:** Distribution of bird species among different groups, based on species-specific ecological patterns (habitat, diet), body size, and social behavior (flocking *vs.* non flocking species). See also [10].

*ID group*	*Species group*	*Some examples*
1	Grebes and divers	*Tachybaptus ruficollis, Podiceps nigricollis, Gavia immer*
2	Cormorant, pelicans, swans and geese	*Phalacrocorax carbo, Cignus olor, Anser anser*
3	Herons, storks, flamingoes	*Ardea cinerea, Casmerodius albus*
4	Ducks, pheasants, rallids	*Anas platyrhynchos, Tadorna tadorna, Phasianus colchicus*
5	Birds of prey – large	*Buteo buteo, Circus aeruginosus*
6	Birds of prey – small	*Falco peregrinus, Falco tinnunculus*
7	Seabirds – large	*Larus michahellis, Larus argentatus*
8	Seabirds – small	*Chroicocephalus ridibundus, Sterna hirundo*
9	Waders	*Charadrius alexandrinus, Recurvirostra avosetta, Tringa totanus*
10	Doves	*Columba livia, Streptopelia decaocto*
11	Owls	*Athene noctua, Tyto alba*
12	Swifts and swallows	*Apus apus, Hirundo rustica*
13	Corvids	*Corvus cornix, Pica pica*
14	Non-flocking passerines and bats	*Erithacus rubecula, Motacilla alba. Turdus merula, Nyctalus noctula*
15	Flocking passerines	*Sturnus vulgaris*
16	Small mammals (<10 kg)	*Vulpes vulpes*
17	Large mammals (>10 kg)	*Dama dama*

**Table 4:** Categories of the Effect On Flight (EOF) provoked by wildlife strike events.

EOF Value	Category	Description
1	None	None
2	Minor	Delay
3	Substantial	Precautionary landing, aborted take-off
4	Serious	Engine(s) shutdown, forced landing, vision obscured
5	Catastrophic	Damage sustained makes it inadvisable to restore aircraft

The previously described modifications were introduced and lead to the following set of equations:

Equation 1:
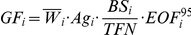



Equation 2:
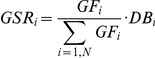



Equation 3 
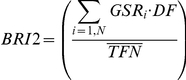
which represent, respectively, the historical risk associated to a species, or Group Factor (GF_i_), the actual Group Specific Risk (GSR_i_), and the second version of the index BRI2.

In Eq. 1–3, *i* indicates a species group (see Table 3), *N* is the group total, 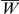 the average weight of the i^th^ group, *Ag* the group specific aggregation index, *BS* is the mean value of impacts recorded per year, *TFN* is the mean value of flights per year and 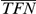 its monthly average. *DBi* represents the mean daily number of birds of the i^th^ group, and *DF* is the mean daily flight traffic calculated on a monthly basis. Note that, *EOF* was defined according to the possible effects, from no effect to airplane damage beyond repairability, according to the 5 level ranking proposed in Table 4. Furthermore, with respect to the original work of Soldatini, et al.[10], Ag is now computed as the average number of birds observed within a group over the entire wildlife dataset, with a minimum Ag value equal to 1.

The index was applied to all sites using mean daily values of bird abundance (DB) and flights (DF) and BRI2 scores obtained for those airports located in the same geographic region, namely airports E *vs.* G and F *vs.* H, were compared by using Spearman correlation.

As the occurrence of incomplete data on wildlife strikes (like, e.g., non-determined species) is frequent, the reliability of BRI2 was verified by randomly excluding an increasing number of observations (10%, 20%, 30%, 40%, and 50%) from the strike dataset for airport G, where the longer time series was available, and computing the index for the year 2010. Results obtained with the full dataset and the reduced ones were compared by means of the Wilcoxon Signed Rank test [28]. This analysis was performed with the twofold objective of assessing the robustness of the Group Factor and define an acceptable limit for the amount of undetermined wildlife strike events.
